# Real-life management of patients with mild cognitive impairment: an Italian survey

**DOI:** 10.1007/s10072-024-07478-9

**Published:** 2024-03-25

**Authors:** Laura Bonanni, Annachiara Cagnin, Claudia Carrarini, Giancarlo Logroscino, Camillo Marra, Innocenzo Rainero

**Affiliations:** 1 Neurology Clinic, Department of Medicine and Aging Sciences, University G. D’Annunzio of Chieti-Pescara, Chieti, Italy; 2 Neurology Clinic, Department of Neuroscience and Padova Neuroscience Center (PNC), University of Padova, Padua, Italy; 3 Department of Neuroscience, Catholic University of Sacred Heart, Rome, Italy; 4 IRCCS San Raffaele, Rome, Italy; 5 Center for Neurodegenerative Disorders and Brain Aging Ospedale Panico, Università Di Bari Tricase (LE), Bari, Italy; 6 Memory Clinic, Department of Neuroscience, Sensorial Systems, and Chest, Fondazione Policlinico A. Gemelli, IRCCS, Rome, Italy; 7 Department of Neuroscience, Catholic University of the Sacred Heart, Rome, Italy; 8 Aging Brain and Memory Clinic, Department of Neuroscience “Rita Levi Montalcini”, University of Torino, Turin, Italy

**Keywords:** Mild cognitive impairment, Survey, Early diagnosis, Real-life, Mild neurocognitive disorder

## Abstract

**Background:**

Mild cognitive impairment (MCI) is a syndrome with heterogeneous underlying causes and different rates of disease progression, whose clinical heterogeneity leads to a wide variation in diagnostic and therapeutic approaches in clinical practice. The lack of uniform practical recommendations on diagnostic workup and treatment for MCI patients hinders optimal management of these patients, worsening their prognosis. Standardized guidelines for the investigation and follow-up of MCI are therefore urgently required.

**Aim:**

Aim of our study was to assess the diagnostic and therapeutic approach to MCI patients in the setting of Italian Memory Clinics.

**Methods:**

A survey was delivered to a sample of Italian neurologists through two different phases: a first exploratory phase recording general information about the usual clinical management of patients with MCI, and a subsequent operative phase assessing the practical diagnostic and therapeutic decisions taken in a real life setting to manage subjects with MCI.

**Results:**

A total of 121 neurologists participated to the first phase of the survey and 203 patients were enrolled in the second phase. Information gathered in the first phase of the survey highlighted a non-uniform use of diagnostic criteria and procedures for MCI, as well as a very heterogeneous therapeutic strategy among Italian neurologists. In the second phase, recorded data on diagnostic and therapeutic approach confirmed the large variability observed in the first phase of the survey.

**Conclusions:**

The results of our study reflect a suboptimal management of MCI patients in Italy and highlight the need of standardized diagnostic and therapeutic approaches for this condition.

**Supplementary Information:**

The online version contains supplementary material available at 10.1007/s10072-024-07478-9.

## Introduction

Mild cognitive impairment (MCI) is a neurological construct that refers to a condition in which an objective cognitive impairment on neuropsychological tests is present in the absence of significant impact on daily living activities. MCI represents an intermediate state between normal cognition and dementia, but in some cases, it is not associated to any pathological process [1, 2]. The condition of MCI corresponds to ‘mild neurocognitive disorder’ in the DSM-5 [3]. MCI prevalence and incidence are age-related with prevalence in the range from 6 to 25% [4, 5] and incidence reaching approximately 60/1000 person-years in the over 85 s [6, 7].

Different diagnostic criteria and subtypes of MCI have been proposed and modified over time [8], reflecting the evolving recognition of MCI as a possible early disease state in the Alzheimer’s disease (AD) continuum [9], as well as in the continuum of other neurodegenerative diseases and vascular cognitive impairment [10]. Annual rates of MCI progression are variable, estimated between 8 and 15% in studies lasting over 5 years [11].

Since MCI is a syndrome with heterogeneous underlying causes and different rates of disease progression, this clinical heterogeneity leads to a wide variation in diagnosis, prognosis and therapeutic approaches [2, 12]. A recent work by Boccardi et al. proposed a consensus biomarker-based diagnostic algorithm, to overcome the variation in diagnostic work-up in neurocognitive disorders [13]. Nevertheless, there is currently no unanimously accepted practical guidance on diagnostic workup and treatment for MCI patients. The lack of widely used recommendations for the treatment of MCI underlines the need to integrate available evidence into guidelines to improve the management of this condition [8].

Considering the evolving importance of identifying the prodromal stages of neurodegenerative diseases leading to dementia and the proposed Italian guidelines for a biological diagnosis of MCI [13], we aimed at assessing the routine diagnostic work-up of MCI and treatment options in the setting of Italian Memory Clinics. To this purpose, a survey was delivered to a sample of Italian neurologists to evaluate the current clinical approach to MCI patients, through two different phases: a first exploratory phase enquiring about general information on the clinical management of patients with MCI, and a follow-up operative phase assessing in a real life setting the diagnostic and therapeutic decisions leading to diagnosis and management of subjects with MCI.

## Methods

A Scientific Board, including six neurologists with expertise in the management of patients with MCI, was appointed to supervise an educational project aimed at improving the clinical approach to MCI in everyday clinical practice.^1^

A total of 17 Italian centers, equally distributed among North, Center and South Italy, were invited to take part in the project (Supplementary material 1). The selected centers were deliberately chosen based on their involvement in the management of patients with MCI. All of them accepted to participate in the survey. Each center identified around 10 clinicians to be involved in two series of local meetings aimed at presenting current literature on the management of MCI and dementia and at sharing real-life experiences. Selected clinicians were deliberately chosen based on their experience in the management of patients with MCI. Both neurologists belonging to Centers for Cognitive Disorders and Dementia (CDCDs) and general neurologists were included.

Involved clinicians were administered an anonymous online survey (through a dedicated web platform) including two phases: i) a first “exploratory” phase (completed before the first series of meetings) enquiring the current clinical approach to patients with MCI, in terms of diagnosis, treatment and follow-up visits; ii) a second “operative” phase (completed before the second series of meetings), consisting in the systematic recording of MCI patients’ data during every day clinical practice for a period of 3 months. The results of the exploratory and operative phases of the survey were analyzed and discussed during the first and second series of meetings, respectively.

The exploratory phase of the survey included 29 items with dual or multiple-choice responses, evaluating five main areas: i) diagnostic work-up of patients with cognitive impairment (diagnostic criteria and differential diagnosis); ii) diagnostic tools (neuropsychological tests, laboratory exams, biomarkers, neuroimaging, genetic screening); iii) assessment of comorbidities (including psychiatric disturbances); iv) pharmacological and non-pharmacological treatments; v) follow-up visits and psychological support to caregivers (Supplementary material 2).

The operative phase of the survey consisted in recording data of MCI patients managed in everyday clinical practice (up to 10 patients for each clinician), covering four main sections: i) demographics; ii) MCI diagnosis (including performed neuropsychological, laboratory, and neuroimaging tests); iii) therapy (including prescribed pharmacologic and non-pharmacologic interventions); iv) timing of follow-up (Supplementary material 3).

## Ethical approval

Although involving human subjects, the present study included no intervention and did not affect routine clinical practice. Clinical data were anonymously recorded and presented in aggregated form. No ethical approval was requested to conduct the study.

## Results

### First phase: exploratory survey

A total of 121 neurologists participated to the first phase of the survey. Most of them (62.5%) were employed in hospital institutions, 26.3% worked at the community level, and a minority as university personnel (6.9%) or as private practitioners (4.3%). Most neurologists (88.6%) reported to visit often (several times per month) patients with MCI, and 83.1% reported to use the diagnostic category of MCI. However, the use of alternative diagnostic criteria (i.e., cognitive impairment-no dementia, age-associated memory impairment) was also reported to be used by 40.2% of participants.

Most clinicians reported to further specify MCI diagnosis, as either amnestic (aMCI) or non-amnestic (naMCI) MCI (87.5%), or as single- or multiple-domain MCI (72.2%). Almost all clinicians (98.5%) reported to discuss with patients the possibility that MCI may evolve to dementia. Dementia diagnosis was made according to DSM-5 (69.4%) or other diagnostic criteria (55.5%). However, 40% of clinicians did not diagnose prodromal AD or MCI due to AD and more than half of them (54.4%) seldom used the proposed ATN scheme for a biological diagnosis of AD [9] (only 22.8% often used them).

Diagnostic assessments are summarized in Table 1. Regarding neuropsychological investigations, the Mini-Mental State Examination (MMSE) was the neuropsychological test most frequently used for screening (93.3%), followed by Montreal Cognitive Assessment (MoCA) (57.1%). In MCI patients, 70.4% of participants performed second level neuropsychological assessments.

**Table 1:** Diagnostic assessments usually performed by physicians participating to the first phase of the survey (*n* = 121)

Assesment	%
Neuropsychological tests	
- MMSE	93.3
- MoCA	57.1
- Second level neuropsychological assessments	70.4
Laboratory tests	
- Blood cell count	90.1
- Vitamin B12	100
- Folates	97.1
- Thyrpoid hormones	95.7
- Hepatic function indexes	78.8
CSF analysis	
- Routinely recommended	26.7
- Rarely recommended	53.3
- Never recommended	19
Instrumental investigations	
- Brain MRI	97.5
- Brain CT	62,4
- Brain FDG-PET	52.5
- PET with amyloid tracer	45.2
- EEG	50,0
Genetic test	
- Often recommendend	9.8
- Rarely recommended	59.1
Assessment of comorbidities	100
Clinical follow-up visits	
- Every 3 months	26.8
- Every 6 months	65.8
- Every 12 months	7.4

Almost the totality of participants reported to routinely perform laboratory tests. Cerebrospinal fluid (CSF) analysis and genetic test were rarely recommended by around half of clinicians (53.5 and 59,1%), respectively).

**Fig. 1:**
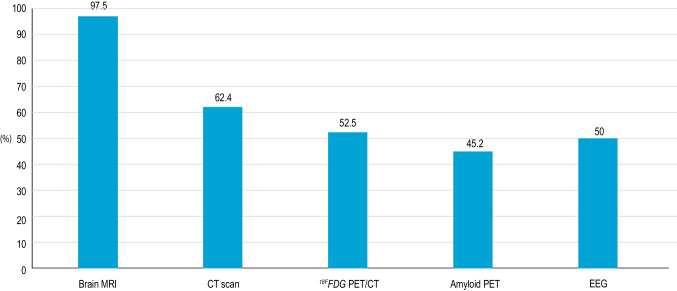
Depicts which neuroradiological and electrophysiological exams are commonly used for MCI diagnosis in clinical practice (exploratory phase of the survey). *Abbreviations: MRI* = *magnetic resonance imaging; CT* = *computed tomography;*^*18F*^*FDG* = ^*18F*^*fluorodeoxyglucose; PET* = *positron emission tomography; EEG* = *electroencephalogram*

As regards instrumental investigation, most clinicians (97.5%) recommend brain magnetic resonance imaging (MRI), while 52.5% prescribed fluorodeoxyglucose positron emission tomography (FDG-PET) and 45.2% PET with amyloid tracer (Fig. 1 and Table 1).

Clinical follow-up visits were mostly recommended every 6 months (65.8%).

**Fig. 2:**
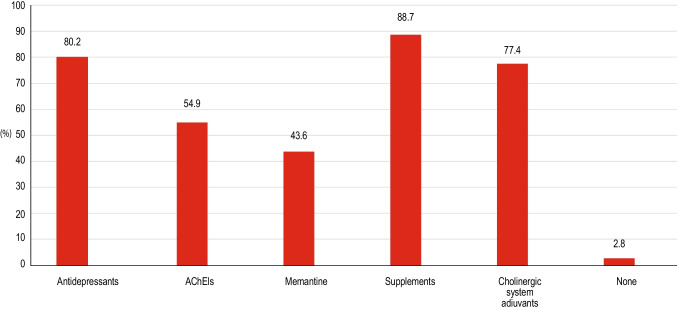
Different first-line treatments used in clinical practice for MCI patients in the first phase of the survey (exploratory phase). *Abbreviations: AChEIs* = *acetylcholinesterase inhibitors*

Prescribed pharmacological therapies are shown in Fig. 2. Most clinicians used supplements (88.7%), anti-depressive drugs (80.2%) or cholinergic system adjuvants (77.4%). Cholinesterase inhibitors were prescribed by 54.9% of clinicians and memantine by 43.6%. Only 2.8% did not prescribe any drugs. Selected MCI subjects were proposed to participate to trials on experimental pharmacological therapies by 47.2% of clinicians.

Regarding non-pharmacological interventions, all clinicians recommended lifestyle changes and monitoring of vascular risk factors and most of them provided nutritional advice (69.4%). Most clinicians also recommended cognitive stimulation interventions (90.4%), rehabilitation/occupational therapies (94.5%), and participation to daycare centers (50%). Concerning recommendations on driving license, 63.4% of clinicians did not discourage the driving of vehicles. Regarding patient’s family/caregivers, 46.4% of clinicians provided supporting activities to caregivers through psychological interventions.

### Second phase: operative survey

All neurologists involved in the first phase of the survey participated to the second phase as well. A total of 203 patients with MCI (mean age 72 years; 52% male) were evaluated and included in the operative survey (91 from North, 21 from Center and 91 from South Italy). Clinical features of enrolled patients and performed investigations are reported in Table 2. aMCI and naMCI were diagnosed in 69.4% and 30.6% of patients, respectively. Among patients with aMCI, 67.6% were classified as single-domain, whereas, among naMCI patients, 84.2% had a multiple-domain impairment. Attention was the most frequently affected domain (65%) in naMCI, followed by memory (51%), language (47.6%) and visuospatial functions (46%). Prodromal AD or MCI due to AD was diagnosed in 32% of patients. Psychiatric comorbidities were more prevalent in younger patients (50–65 years) (Table 2).

**Table 2:** Clinical features of patients enrolled in the second phase of the survey (*n* = 203, mean age 72 years)

Clinical characteristics	%
Gender	
- Male	52
- Female	48
Age group	
- 50–65 years	16.9
- 66–75 years	52.1
- 76–85 years	27.8
- > 85 years	3.2
Diagnosis	
- aMCI	69.4
- single domain impairment	67.6
o multiple-domain impairment	32.4
o 50–65 years	66.6
o 66–75 years	70
o 76–85 years	73.4
o > 85 years	57.1
- naMCI	30.6
o single domain impairment	15.8
o multiple-domain impairment	84.2
o 50–65 years	33.4
o 66–75 years	30
o 76–85 years	26.6
o > 85 years	42.9
- Prodromal AD/MCI due to AD	32
o 50–65 years	35.8
o 66–75 years	28.3
o 76–85 years	31.2
o > 85 years	14.2
Psychiatric comorbidities	
- mood disturbances	40.8
o 50–65 years	53.8
o 66–75 years	37.5
o 76–85 years	40.6
o > 85 years	14.2
- anxiety disorders	24.1
o 50–65 years	30.7
o 66–75 years	25.8
o 76–85 years	17.18
o > 85 years	0
- substance abuse	1.4
o 50–65 years	0
o 66–75 years	0.8
o 76–85 years	1.5
o > 85 years	0
Neuropsychologic test used for screening	
- MMSE	95.1
- MoCA	38.4
- Additional tests	58.1
Instrumental investigations performed to confirm diagnosis	
- Brain MRI	80
- Brain CT	33
- Brain FDG-PET	21.1
- PET with amyloid tracer	5.9
- EEG	22.6
Use of genetic tests	3.4

Regarding neuropsychological test screening, MMSE was performed in 95.1% of patients, with a mean score of 24.82, whereas MoCA was performed in 38.4% of patients, with a mean score of 22.06. Additional neuropsychological tests were performed in 58.1% of patients (Table 2).

Among instrumental diagnostic examinations, MRI was performed in 80% of patients, followed by computed tomography (CT) (33%), electroencephalogram (EEG) (22.6%), FDG-PET (21.1%) and PET with amyloid tracer (5.9%). Genetic tests were performed in 3.4% of patients (Table 2).

**Fig. 3:**
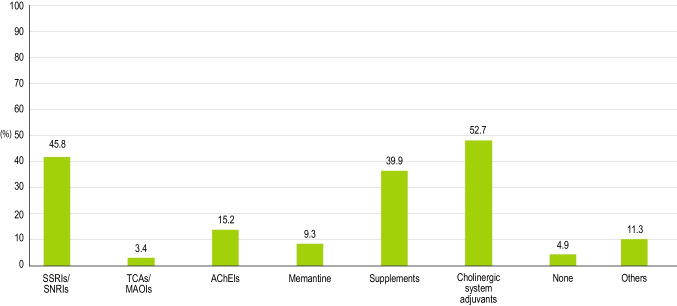
The percentages of different pharmacological therapies prescribed for MCI patients in the second phase of the survey (operative phase). *Abbreviations: SSRIs* = *selective serotonin reuptake inhibitors; SNRIs* = *serotonin and norepinephrine reuptake inhibitors; TCAs* = *tricyclic antidepressants; MAOIs* = *monoamine oxidase inhibitors; AChEIs* = *acetylcholinesterase inhibitors*

Newly prescribed pharmacologic therapy included cholinergic system adjuvants (52.7%), anti-depressing agents (selective serotonin reuptake inhibitors [SSRIs] and serotonin and norepinephrine reuptake inhibitors [SNRIs], 45.8%), supplements (39.9%), cholinesterase inhibitors (15.2%), memantine (9.3%), tricyclic anti-depressants or MAO inhibitors (3.4%); 4.9% of patients were untreated (Fig. 3). In addition, treatments to control cardiovascular risk factors were prescribed to 67.9% of patients.

**Fig. 4:**
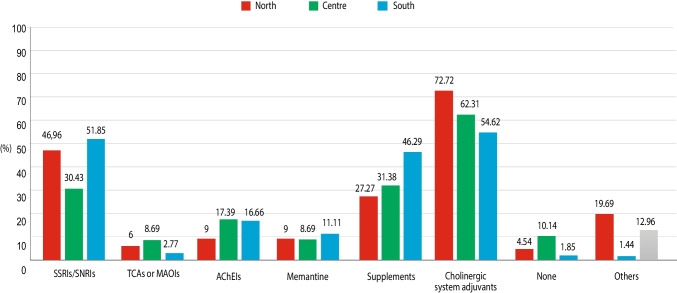
The percentages of different pharmacological therapies prescribed for MCI patients in the second phase of the survey (operative phase), stratified for geographical regions

After stratifying results according to the geographical area (North, Centre, and South), absence of treatment was more prevalent in the Centre (10.14%) compared with North (4.54%) and South (1.85%). The use of supplements was more prevalent in southern (46.29%) compared with northern (27.27%) or central (31.88%) regions. The use of cholinergic system adjuvants was more prevalent in northern (72.72%) than in central and southern regions (62.31 and 54.62%, respectively). Prescription of antidepressant agents (SSRIs/SNRIs) was more prevalent in the South (51.85%) than in the North (46.96%) or in the Centre (30.43%), whereas prescription of cholinesterase inhibitors was more frequent in the Centre and South (17.39 and 16.66%, respectively) than in the North (9.0%). No substantial differences among regions were observed in the prescription of memantine, tricyclic anti-depressants or MAO inhibitors (Fig. 4).

Regarding non-pharmacological treatments, rehabilitation/behavioral interventions were prescribed to 20.6% of patients and dietetic advice was provided to 49.7% of patients. Additionally, 50.2% of patients were suggested to avoid driving.

Follow-up visits were planned every 3 (29.6%), 6 (66%) or 12 months (4.4%).

## Discussion

MCI is a common condition in the aging population that progresses to dementia in a significant proportion of cases. Still this condition is underdiagnosed and undertreated. At present, few studies evaluated the opinion and the treatment strategies of experts and general practitioners in the diagnosis and treatment of these patients. Our survey investigated, with a two-phase strategy, how Italian neurologists diagnose and treat this condition, providing a few interesting observations. First of all, in accordance with previous studies [2, 8], in the first phase of the survey we found that the use of diagnostic criteria for MCI is not uniform, resulting in a significant delay in both identification and treatment of cognitive impairment. Moreover, although almost all clinicians inform patients about the possible evolution of MCI into dementia, only 50% of neurologists diagnose prodromal AD or MCI due to AD. MMSE is still the preferred screening tool (used by 93.3% of clinicians), whereas MoCA is used more rarely (57.1%), even though MoCA widely showed its superiority in detecting MCI condition [14]. However, the use of second level neuropsychological tests in MCI patients is common.

Moreover, whereas standard laboratory tests are routinely prescribed by all clinicians, diagnostic biomarkers and genetic tests are seldom used for the diagnosis of cognitive impairment in clinical practice, probably due to a lack of awareness of their role in the absence of a disease modifying therapy. In addition, a quite variable approach was observed in the use of neuroimaging and electrophysiology for the confirmation of MCI diagnosis: whereas MRI is prescribed by most clinicians (97.5%), CT, FDG-PET, and amyloid PET are also taken into consideration by a large proportion of neurologists (62,4, 52,5 and 45,2%, respectively), and EEG is used by half of them (50%).

Therapeutic approach to MCI is still a matter of intense debate, as there are currently no drugs that have been approved for treating this condition. Accordingly, our study shows that first-line therapeutical strategy among Italian neurologists, although heterogeneous, is based on the preferential use of supplements, cholinergic system adjuvants and antidepressant drugs respect to cholinesterase inhibitors and memantine, which play a marginal role. The potential use of experimental drugs is taken into consideration by half of the clinicians. These figures partly reflect the available evidence on the efficacy of different interventions for the management of MCI: while the infrequent use of cholinesterase inhibitors and memantine is in accordance with the absence of documented efficacy of these drugs in MCI subjects, the widespread use of supplements and cholinergic adjuvants probably reflects the attempt reported in literature to enhance cognitive function by interventions based on various forms of choline supplementation [15]. A 2005 Cochrane Review of 14 double-blind, placebo-controlled studies using citicoline for cognitive impairment due to chronic cerebral disorders showed “some evidence that citicoline has a positive effect on memory and behavior in at least the short to medium term and especially in patients suffering of cognitive deficits associated with cerebrovascular disorders.” [16]. In the last two decades, a large number of studies have investigated the effects of citicoline supplementation in modulating cognitive functions in several neurological diseases, like Alzheimer’s disease, Parkinson’s disease, MCI and vascular cognitive impairment [15, 17–22]. More recently, a meta-analytic study including 6 studies on citicoline supplementation in patients with Alzheimer’s disease, MCI and post-stroke dementia published between 2010 and 2022, suggested that citicoline has a positive effect on cognitive functions, although the general quality of the included studies was poor [23]. Studies in experimental animals have shown that citicoline modulates both adrenergic and dopaminergic metabolism in the brain [24]; moreover, it seems to enhance cerebral metabolism, and to exert neuroprotective actions in animals and humans [18, 19, 25]. These data, together with the registered indications of citicoline in Italy for the treatment of neurological and cognitive disorders associated to cerebrovascular accidents or traumatic cerebral lesions [26], might explain the frequent use of this drug observed in our survey.

On the other hand, there is a strong rationale for treatment of neuropsychiatric symptoms, which are common in MCI patients and may be associated with greater functional impairment and progression to Alzheimer’s dementia [27]: in this respect, the wide use of antidepressant drugs for the management of MCI recorded in the first phase of the survey may reflect the high prevalence of mood and behavioral disorders in these patients, as documented in the operative phase as well. As regards the use of antidepressant drugs, however, it should be noted that their effect on cognition in the elderly is controversial, with some studies suggesting harm and others protection [28, 29]. Although these drugs are widely prescribed as a first-line treatment of depression in both MCI and dementia, the NICE guidelines 2018 do not suggest the routine treatment of depression and anxiety with antidepressants in people with dementia [30]. Thus, psychosocial interventions should be tried first, considering antidepressant use only in MCI patients with major depression or in those for whom depression is regarded by the clinician as the main determinant of cognitive impairment.

Non-pharmacological interventions (i.e., lifestyle changes, cognitive stimulation, and rehabilitation/occupational therapy) are recommended by the vast majority of physicians, whereas referral to daycare centers is not always prescribed and support to caregivers is provided by almost half of clinicians only.

The second phase of the study was performed to obtain a confirmatory validation, in a real-life setting, of the propositive attitude towards a specific diagnostic and therapeutic pathway declared by clinicians in the first phase of the survey, by recording the actual behavior they had with their own patients in everyday clinical practice. Use of diagnostic cognitive and instrumental tools in clinical practice at some extent corresponded to what observed in the first phase of the survey, with more prevalent use of MMSE than MoCA (95.1 vs 38.4%), prescription of MRI in most cases (80%), whereas CT, FDG-PET, amyloid PET and EEG were limited to a smaller proportion of patients (33, 21.1, 5.9, and 22.6%, respectively). Such data suggest that the propositional attitude towards a more extensive use of MoCA and PET, suggested in the first phase, is applied at a lesser extent of what proposed as ideal in the real diagnostic journey of MCI patients. CSF and genetic tests were performed in a very limited proportion of patients showing a very poor tendency of neurologists not primarily involved in CDCDs to proceed towards a biological diagnosis of AD, which is still far from a widespread implementation in clinical practice. However, other factors such as very advanced age, comorbidities, or frailty, may lead clinicians to skip complex diagnostic procedures such as molecular imaging or CSF analysis of biomarkers, possibly explaining the gap between the awareness of recommended examinations and their application in clinical practice. Also, data on pharmacologic approach confirmed the large variability recorded in the first phase of the survey, with a prominent administration of cholinergic system adjuvants and anti-depressing agents. The heterogenicity in the pharmacological approach was observed also at the regional level, with differences in the use of individual drug classes between Northern, Central and Southern Italy. In contrast with what observed in the first phase of the survey, rehabilitation/behavioral interventions were prescribed to a rather small proportion of selected patients, regardless of age.

Taken together, data collected from both the first and second phase of the survey are indicative of a suboptimal management of MCI patients in Italy, highlighting the need of standardized diagnostic and therapeutic approaches for this condition. Our data are in accordance with a study recently performed in Scotland that explored clinicians’ views around the assessment of MCI in memory clinics and revealed a mixture of approaches in assessing and disclosing test results for MCI [31]. Some clinicians consider the condition as a separate entity among neurodegenerative disorders whereas others find the term unhelpful due to its uncertain prognosis. Clinicians report a lack of specific and sensitive assessment methods for identifying the etiology of MCI in clinical practice. In line with our results, this study demonstrates a broad range of views and therefore variability in MCI assessment in clinical practice, which may impact the management of individuals with MCI [31].

A few strong recommendations regarding MCI patients should be shared among experts in dementia and general practitioners. A Consensus Meeting on MCI held in Manchester (UK) suggested several indications regarding the depth of that investigation, the utility of molecular biomarkers, the blood screening and neuropsychological testing recommended [2]. In addition, there is clear indication that patients with MCI diagnosis should undergo a follow-up visit at least annually and should be offered access to research [2]. Similarly, a recent Report of the Guideline Development, Dissemination, and Implementation Subcommittee of the American Academy of Neurology (AAN) provided a series of detailed recommendations concerning the diagnosis and treatment of MCI [32].

To improve early diagnosis and optimize treatment in Italy, a dedicated outpatient neurological network system should be implemented, connecting primary care with CDCDs. CDCDs should be organized in subregional network according to their specific competences, providing the standard diagnostic pathway for all patients, and access to second level (biomarkers, genetic) investigations for selected patients only. As a part of this service, electronic clinical data recording into a shared database might favor communication between peripheral institutions and reference centers, paving the way towards the implementation of a nationwide clinical network for the management of patients with MCI. In addition, considering the long pathway required for the clinical development of potential disease modifying drugs, neurologists should propose experimental protocols to these patients, to offer them the opportunity to benefit of potentially effective drugs.

## Limitations of the study

Although informative of the level of awareness on the disease among clinicians, the presented data offer a partial view of the actual approach to MCI in Italy, given the limited number of neurologists involved, which cannot be considered totally representative of the national situation. The restriction of the survey to neurologists alone, thereby excluding geriatricians and psychiatrists who are also involved in the identification and care of MCI patients, further limits the generalizability of the study results. Moreover, the small number of patients included in the second phase of the survey makes the representativeness of the sample questionable; therefore, data from the operative phase of the study should be considered less informative than those derived from the exploratory phase. A more comprehensive and systematic assessment, possibly involving a higher number of sites and a larger sample of patients, is required to confirm the observed figures and better define intervention strategies. Nevertheless, the current work provides important information on the actual management of MCI in Italy, based on the results of the survey administered to neurologists involved in the assessment of patients with cognitive impairment, and describes the profile of patients with diagnosed or suspected MCI, based on the data recorded by clinicians during everyday clinical practice.

## Conclusions

Given the heterogeneous nature of MCI, all potentially modifiable factors contributing to the cognitive decline should be identified and addressed, to improve symptoms and possibly delay dementia progression. In this respect, shared guidelines specifically focused on MCI are urgently needed to standardize clinical algorithms for prevention, diagnosis, treatment, and follow-up; on the other hand, dedicated healthcare facilities providing MCI patients with specialized medical personnel and psychologic support, also for caregivers, are crucial to improve the overall management of this condition, as well as patients’ prognosis.

### Supplementary Information

Below is the link to the electronic supplementary material.

Supplementary file1 (DOCX 19 KB)

Supplementary file2 (DOCX 25 KB)

Supplementary file3 (DOCX 23 KB)

## Footnotes

1 The members of the Scientific Board are the authors of the present paper.
