# Supplementary material for: Real-life management of patients with mild cognitive impairment: an Italian survey
Source: Neurol Sci. 2024 Mar 25;45(9):4279–89. doi: 10.1007/s10072-024-07478-9 (PMC11306749; doi:10.1007/s10072-024-07478-9)
Supplement: Supplementary file 1 — Supplementary file1 (DOCX 19 KB) [file 10072_2024_7478_MOESM1_ESM.docx]

1. Ospedale Torrette, Ancona
2. Ospedale Santa Croce, Moncalieri (SI)
3. CDCD ASL 3, Genova
4. Villa Scassi, Genova
5. H Civile - Stroke Unit, Padova
6. H Civile - Disturbi Cognitivi, Padova
7. USL Umbria 1, Perugia
8. Un Pisa, Pisa
9. Niguarda, Milano
10. Policlinico Torvergata, Roma
11. ASL Pomigliano d'arco, Napoli
12. PTA ASP PALERMO CENTRO, Palermo
13. Ospedale Monopoli, Monopoli (BA)
14. Ospedale SS Annunziata, Chieti
15. ARNAS Garibaldi, Catania
16. H Reg. Pugliese-Ciacciò, Catanzaro
17. ASP Caltanissetta, Caltanissetta
