# Supplementary material for: Real-life management of patients with mild cognitive impairment: an Italian survey
Source: Neurol Sci. 2024 Mar 25;45(9):4279–89. doi: 10.1007/s10072-024-07478-9 (PMC11306749; doi:10.1007/s10072-024-07478-9)
Supplement: Supplementary file 2 — Supplementary file2 (DOCX 25 KB) [file 10072_2024_7478_MOESM2_ESM.docx]

**DEMOGRAPHICS**

**Age**

_____________ years

**Gender**

F M

**Professional activity duration**

__________ years

**Main professional actvity setting**

Hospital University Community Private office

**Professional activity location**

Region ___________

**QUESTIONNAIRE**

1. *In your clinical practice, how often do you visit patients with mild cognitive impairment?*
   1. **Never Yes No**
   2. **Seldom (<1 time/month) Yes No**
   3. **Sometimes (1-2 times/month) Yes No**
   4. **Often (several times/month) Yes No**

1. *Do you use the diagnostic category of MCI (Mild Cognitive Impairment)?*

**Yes No**

1. *Do you use alternative diagnostic criteria (i.e., cognitive impairment-no dementia, age-associated memory impairment)?*

**Yes No**

1. *Do you further specify MCI diagnosis?*
   1. **Amnestic (aMCI) or non-amnestic (naMCI) MCI Yes No**
   2. **Single- or multiple-domain MCI Yes No**
2. *Do you discuss with patients the possibility that MCI may evolve to dementia?*

**Yes No**

1. *Which diagnostic criteria do you use for to diagnose dementia?*
   1. **DSM V Yes No**
   2. **Other international diagnostic criteria Yes No**
2. *In your clinical practice, do you diagnose prodromal AD or MCI due to AD?*

**Often Seldom Never**

1. *In your clinical practice, do you use ATN criteria?*

**Often Seldom Never**

1. *Which of the following neuropsychological investigations do you use for MCI screening?*

**a. MMSE Yes No**

**b. MoCA Yes No**

1. *Do you use second level neuropsychological assessments in patients with MCI?*

**Often Seldom Never**

1. *Which of the following laboratory tests do you routinely perform in patients with MCI (select one or more)*

**a. Vit B12 Yes No**

**b. Folates Yes No**

**c. Thyroid hormones Yes No**

**d. Blood count Yes No**

**e. Liver enzymes Yes No**

1. *Do you recommend cerebrospinal fluid (CSF) analysis in patients with MCI?*

**Often Seldom Never**

1. *Which instrumental investigation do you perform to confirm MCI diagnosis?*

**a. MRI Yes No**

**b. CT Yes No**

**c. FDG PET Yes No**

**d. Amyloid PET Yes No**

**e. EEG Yes No**

*14. Dop you recommend genetic tests (APOE, etc.) in patients with MCI?*

**Often Seldom Never**

1. *Do you assess internal medicine comorbidities in patients with MCI?*

**Yes No**

1. *Do you assess psychiatric comorbidities in patients with MCI?*

**Yes No**

1. *Do you recommend clinic follow-up to MCI patients??*

**Yes**  **No**

*If yes, how often do you visit patients?*

- 1. **Every 3 months Yes No**
  2. **Every 6 monts Yes No**
  3. **Every year Yes No**

1. *Which drugs do you prescribe to MCI patients?*

**a. Antidepressant drugs Yes No**

**b. Cholinesterase inhibitors Yes No**

**c. Memantine Yes No**

**d. Supplements Yes No**

**e. Cholinergic system adjuvants Yes No**

**f. I do not use drugs because of adverse events Yes No**

1. *Do you assess therapy to control cardiovascular risk factors in MCI patients?*

**Yes No**

1. *Do you give behavioral advice to MCI patients?*

**Yes No**

1. *Do you give nutritional advice to MCI patients?*

**Yes No**

1. *Do you recommend cognitive stimulation to MCI patients?*

**Yes No**

1. *Do you suggest experimental drugs to MCI patients?*

**Often Seldom Never**

1. *Do you suggest to avoid driving to MCI patients?*

**Yes No**

1. *Do you think that MCI diagnosis may be useful for the patients and their families?*

**Yes No**

1. *Do you think that MCI diagnosis may cause unjustified stress for the patients and their families?*

**Yes No**

1. *Do you promote supporting activities for caregivers, through group or primary psychologic interventions?*

**Often Seldom Never**

1. *Do you think that occupational/cognitive rehabilitation is useful for the patient?*

**Yes No**

1. *Do you think it’s useful to refer MCI patients to daycare centres?*

**Yes No**
