# Supplementary material for: Real-life management of patients with mild cognitive impairment: an Italian survey
Source: Neurol Sci. 2024 Mar 25;45(9):4279–89. doi: 10.1007/s10072-024-07478-9 (PMC11306749; doi:10.1007/s10072-024-07478-9)
Supplement: Supplementary file 3 — Supplementary file3 (DOCX 23 KB) [file 10072_2024_7478_MOESM3_ESM.docx]

**GENERAL DATA**

PATIENT CODE______
AGE ______
GENDER □ M □ F

**MCI DIAGNOSIS**

- □ Mild amnestic cognitive impairment (isolated memory impairment)

□ Single domain mild cognitive impairment

□ Multiple domain mild cognitive impairment

Which cognitive impairment? (select one or more)
□ Memory
□ Attention
□ Language
□ Visuospacial
□ Other (specify) _____

or

- □ Mild non amnestic cognitive impairment (impairment of a cognitive domain other than memory, such as attention, language or executive functions)

□ Single domain mild cognitive impairment
□ Multiple domain mild cognitive impairment

Which cognitive impairment? (select one or more)
□ Memory
□ Attention
□ Language
□ Visuospacial
□ Other (specify) _____

**MCI DIAGNOSIS Part II**

Was prodromal Alzheimer Disease (AD) or MCI due to AD diagnosed?
□ YES
□ NO

Was the MMSE (Mini Mental State Examination) neuropsychological test administered?
□ YES
□ NO
If yes, score: ___ (from 0 to 30)

Was the MoCa (Montreal Cognitive Assessment) neuropsychological test administered?
□ YES
□ NO
If yes, score: ___

Were other neuropsychological test administered?
□ YES
□ NO
If yes, please specify:
□ Frontal function test (e.g., Rey complex figure)
□ Intelligence test (e.g., WAIS, Raven matrices)
□ Memory test (e.g., Recognition memory test)
□ Language test
□ Attention test (e.g., Barrage test)
□ Test of visual perceptual, visuospacial, visual constructive and praxic functions
□ Other (specify) _____________

**DIAGNOSTIC EXAMINATIONS**

Laboratory tests (optional):
Vitamin B12 _ ng/l
Folates _ ng/ml
Tiroxine (TT4) _ nmoli/l
Triiodothyronine (TT3) _ nmoli/l
Hematocrit _ %
Hb _ g/dl
White blood cells _ x10^3/uL
AST _ Ul/l
ALT _ Ul/l

Neurodiagnostic examinations performed to confirm MCI diagnosis (select one or more):
□ MRI
□ CT
□ FDG PET
□ Amyloid PET
□ EEG

Were genetic test performed (e.g., APOE)?
□ YES
□ NO

**COMORBIDITIES**

Psychiatric comorbidities (opzional, select one or more):
□ Mood disorders (depressive disorders, bipolar disorders)
□ Anxiety disorders (generalized anxiety disorder, panic attack, phobia)
□ Obsessive-compulsive disorder
□ Substance use and abuse
□ Other (specify): ________

Internal medicine comorbidities opzional, select one or more):
□ Cardiac
□ Pneumological
□ Metabolic
□ Endocrinological
□ Neurological
□ Neoplastic
□ Gastroenterological
□ Other (specify): _____

**PHARMACOLOGIC AND NON-PHARMACOLOGIC TREATMENT**

Pre-existing therapy (select one or more):
□ Anti-depressants (SSRI/SNRI)
□ Tricyclic anti-depressants or MAO inhibitors
□ Cholinesterase inhibitors
□ Memantine
□ Supplements
□ Cholinergic system adjuvants
□ None
□ Other (specify): ___________

Was the patient treated to control cardiovascular risk factors?
□ YES
□ NO

Was the patient prescribed cognitive/occupational rehabilitation?
□ YES
□ NO

Therapy prescribed at discharge until next control visit (select one or more):
□ Anti-depressants (SSRI/SNRI)
□ Tricyclic anti-depressants or MAO inhibitors
□ Cholinesterase inhibitors
□ Memantine
□ Supplements
□ Cholinergic system adjuvants
□ None
□ Other (specify): ___________

Was nutritional advice given?
□ YES
□ NO

Was behavioral advice given (e.g., suggestion to avoid driving)?
□ YES
□ NO

Planned follow-up visits:
□ Every 3 months
□ Every 6 months
□ Every 12 months
□ None
